# Supplementary figures and images for: Hydrophobicity drives receptor-mediated uptake of heat-processed proteins by THP-1 macrophages and dendritic cells, but not cytokine responses
Source: PLoS One. 2020 Aug 14;15(8):e0236212. doi: 10.1371/journal.pone.0236212 (PMC7428126; doi:10.1371/journal.pone.0236212)

**A**

Uptaked native protein  
by THP-1 M $\phi$

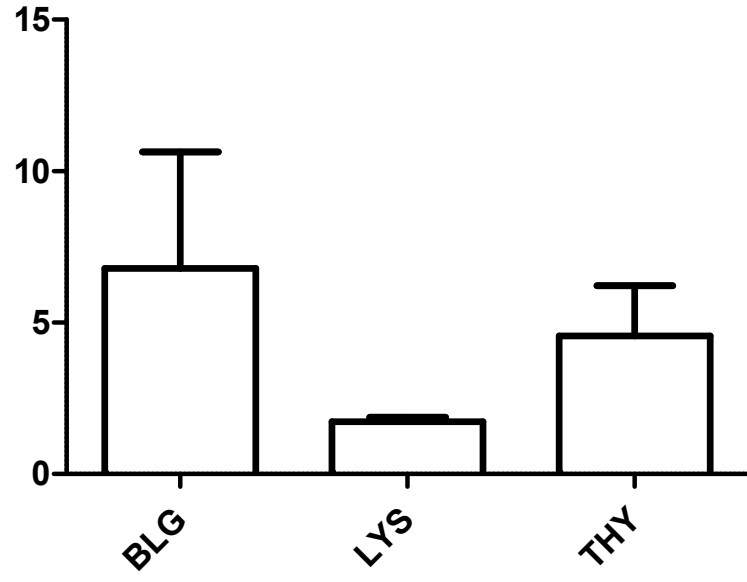**B**

Uptaked native protein  
by THP-1 iDC

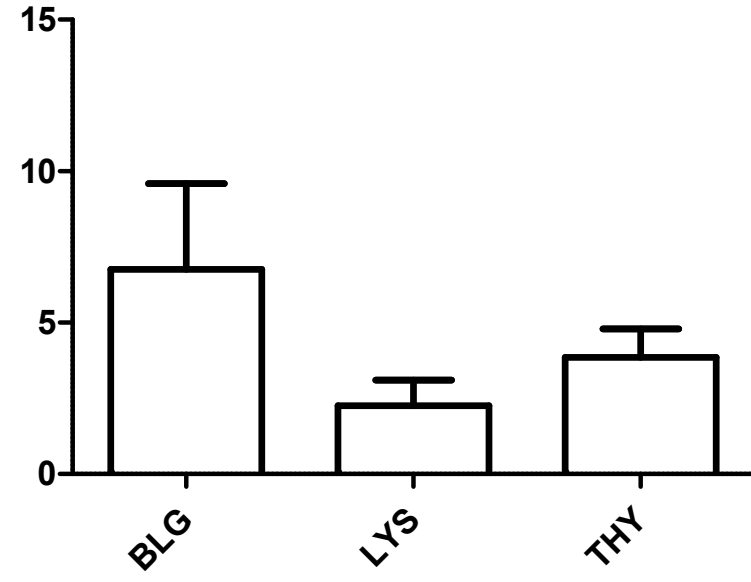

Supplement: S1 Fig — The result represents the mean value ± SD of 4 independent cell experiments. No significant differences have been found using unpaired T-test with Welch’s correction. (PDF) [file pone.0236212.s001.pdf]

A

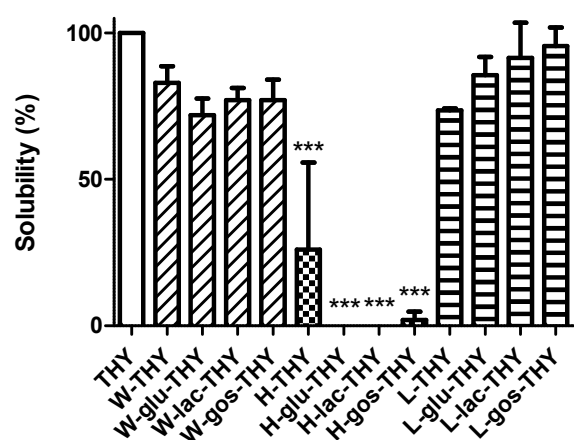

B

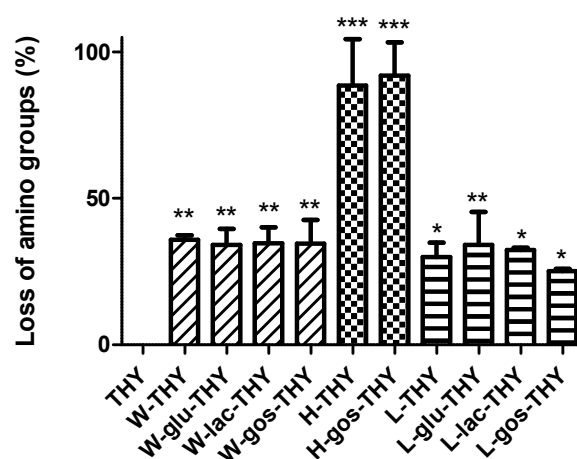

C

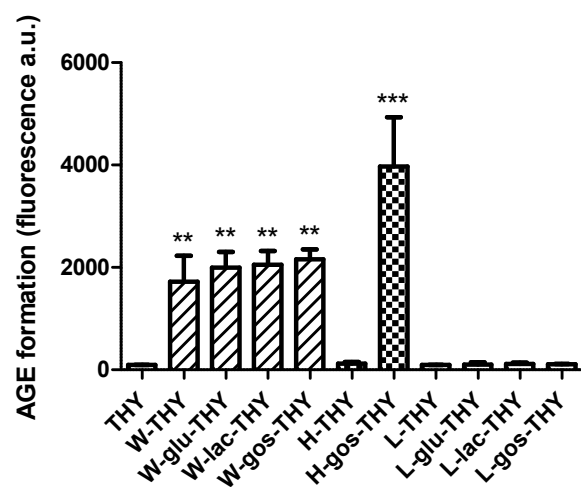

D

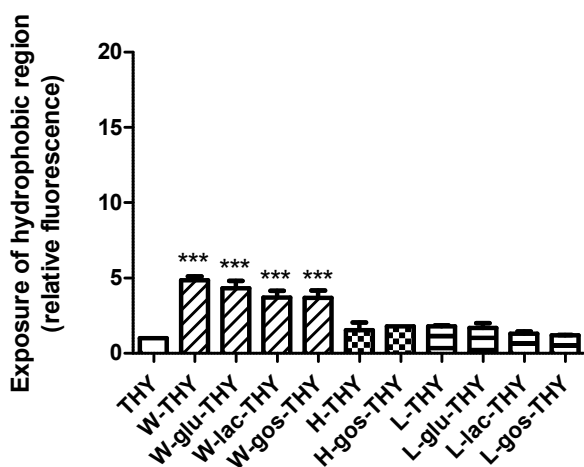

E

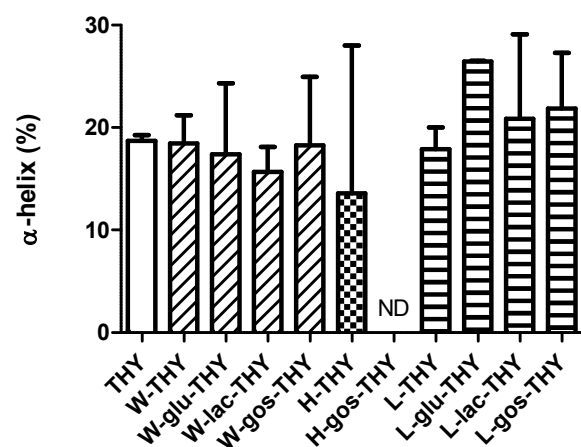

F

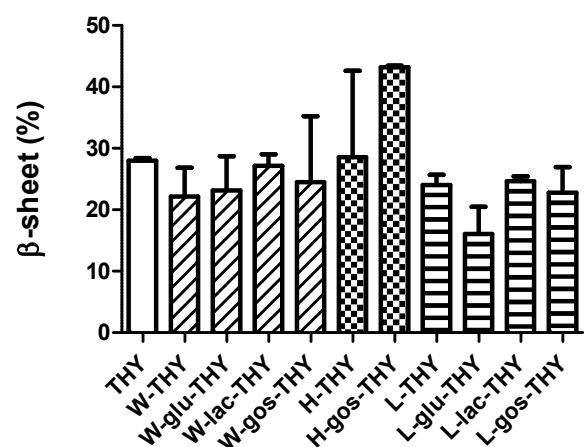

Supplement: S2 Fig — Thyroglobulin (THY) was untreated or heated (W, H or L) in the absence or presence of saccharides (glu, lac or GOS) and a number of physiochemical parameters (i.e., solubility, loss of amino group, AGE-related fluorescence, hydrophobicity, α-helix and β-sheet structure) were measured. The results represent mean values ± SD of n = 4 measurements of 2 independent experiments based on 2 independent sample sets. Statistical differences compared to native THY were calculated with Dunnett’s Test: *p < 0.05; **p < 0.01; ***p < 0.001. ND: not detectable. (PDF) [file pone.0236212.s002.pdf]

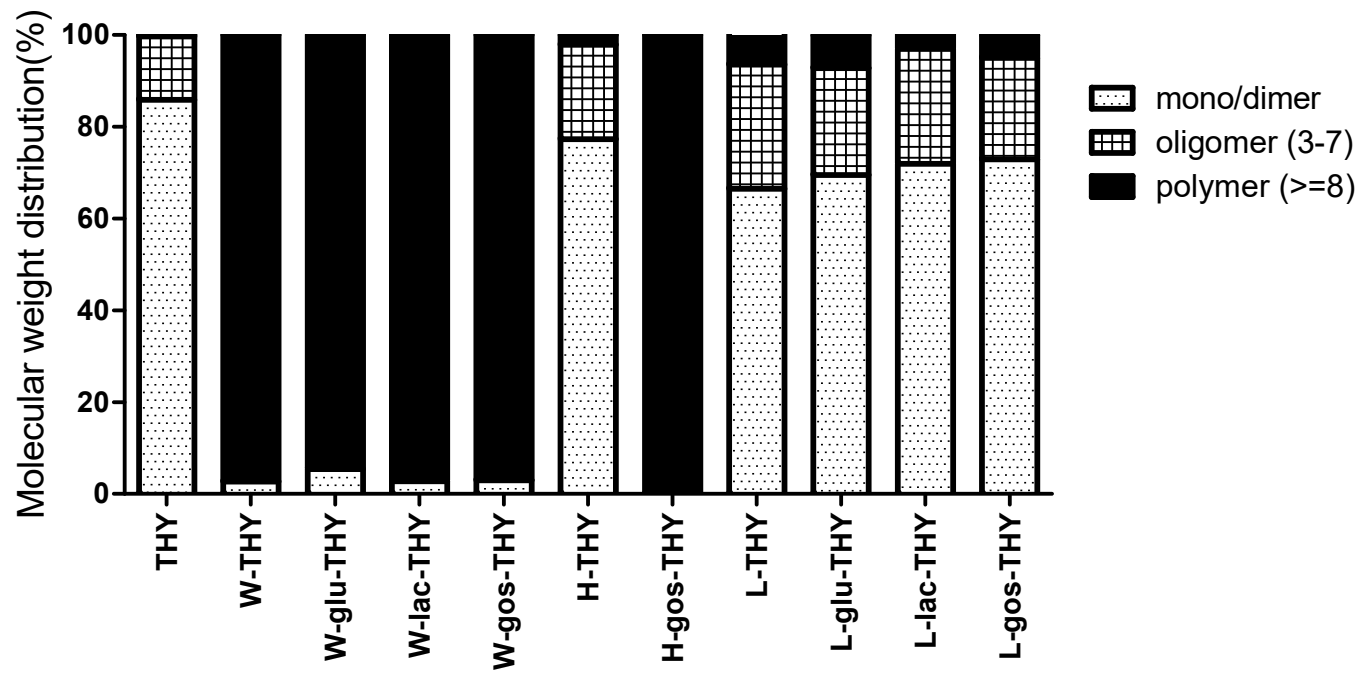

Supplement: S3 Fig — Thyroglobulin (THY) was untreated or heated (W, H or L) in the absence or presence of saccharides (glu, lac or GOS) and aggregation in the soluble fraction was measured using size exclusion chromatography. The data points represent the average values of 2 independent sample sets. (PDF) [file pone.0236212.s003.pdf]

A

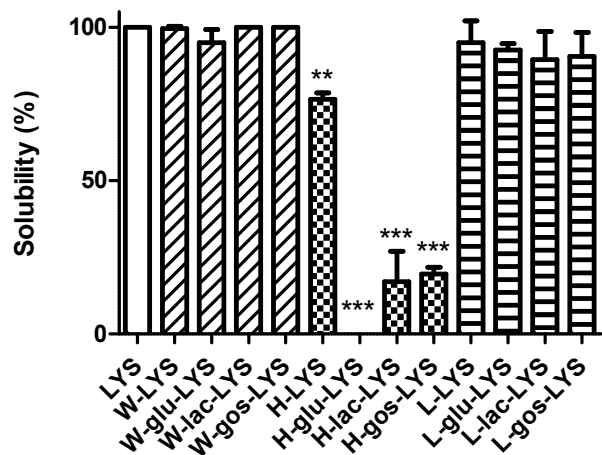

B

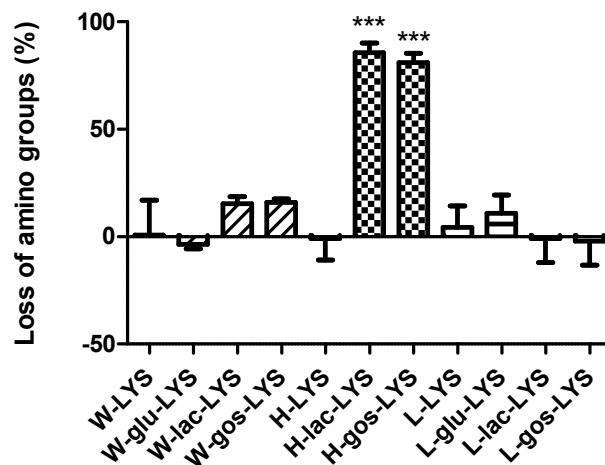

C

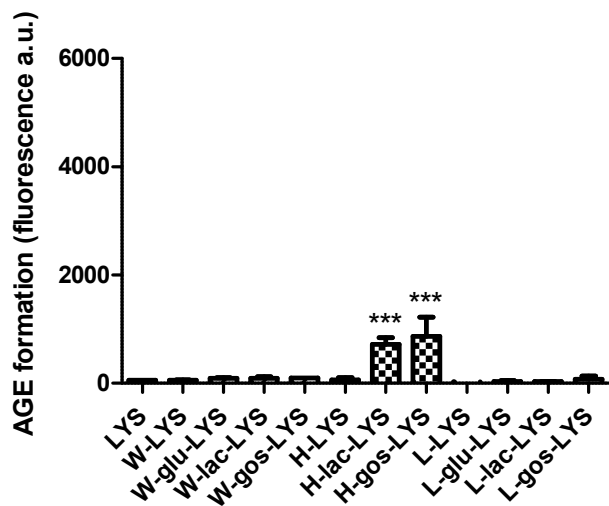

D

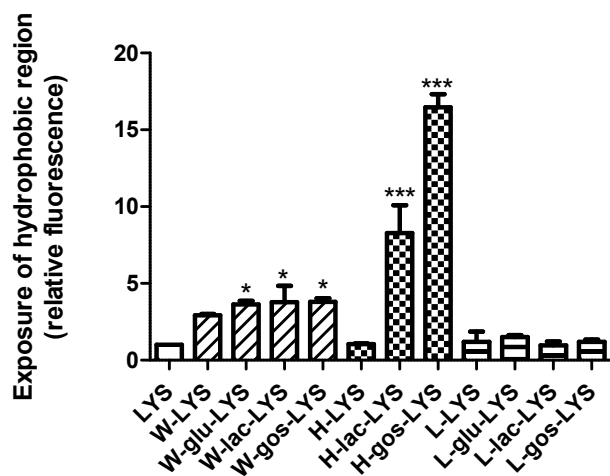

E

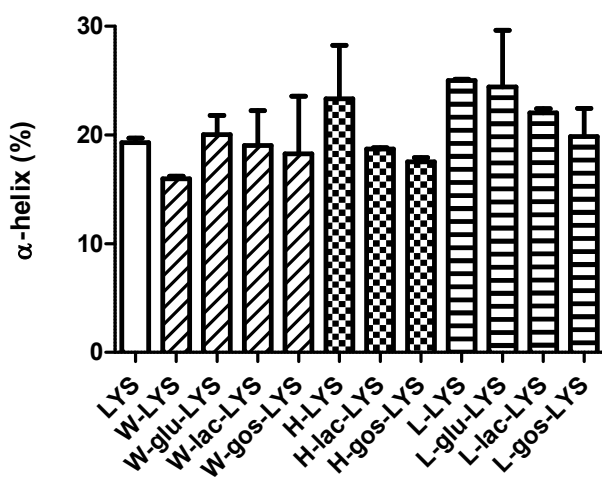

F

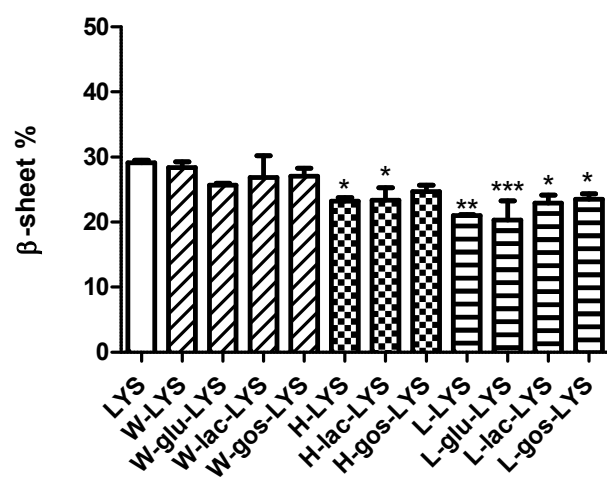

Supplement: S4 Fig — Lysozyme (LYS) was untreated or heated (W, H or L) in the absence or presence of saccharides (glu, lac or GOS) and a number of physicochemical parameters (i.e., solubility, loss of amino group, AGE-related fluorescence, hydrophobicity, α-helix and β-sheet structure) were measured. The results represent the mean values ± SD of n = 4 measurements of 2 independent experiments based on 2 independent sample sets. Statistical differences compared to native LYS were calculated with Dunnett’s Test: *p < 0.05; **p < 0.01; ***p < 0.001. (PDF) [file pone.0236212.s004.pdf]

Bi-plot

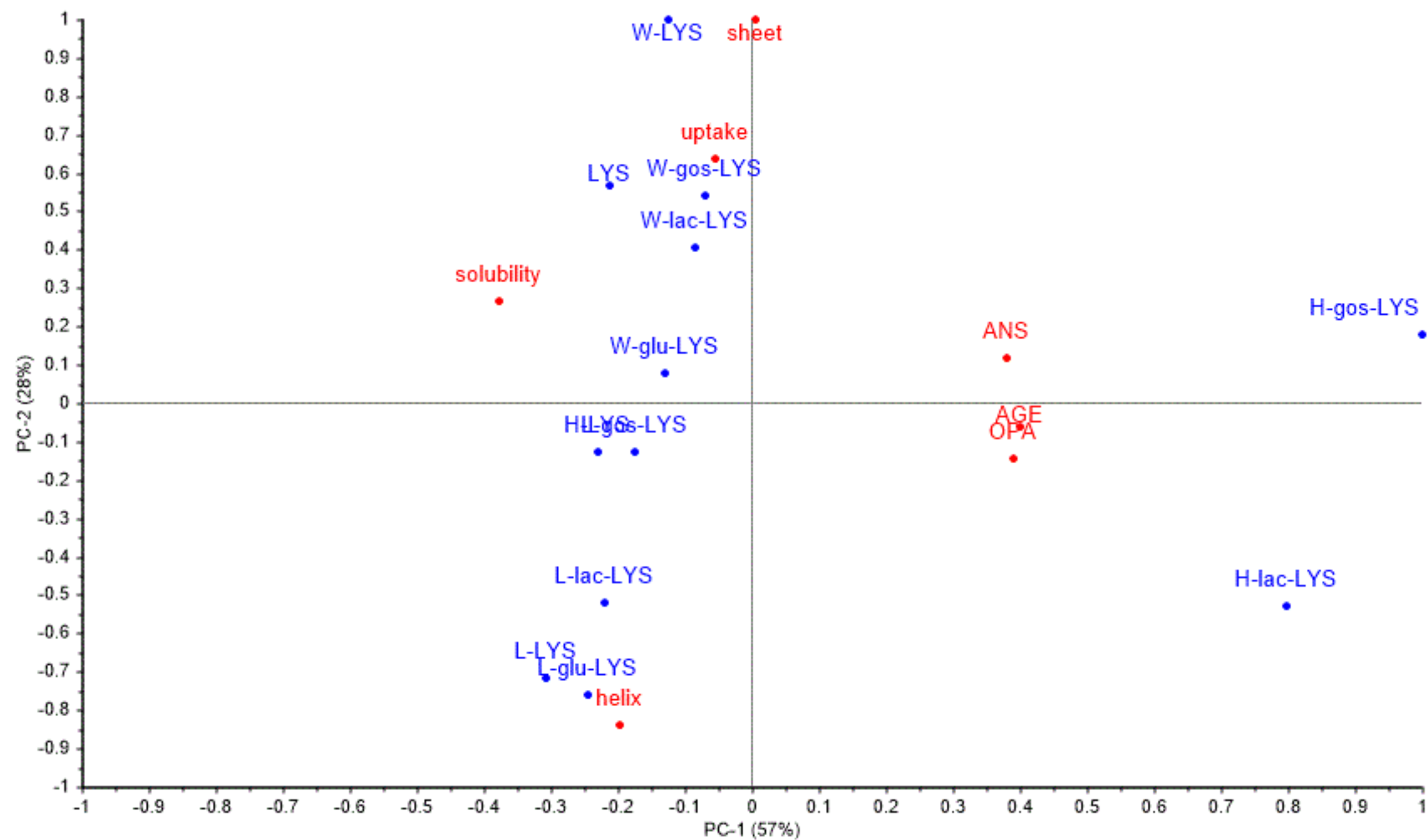

Supplement: S5 Fig — Lysozyme (LYS) was untreated or heated (W, H or L) in the absence or presence of saccharides (glu, lac or GOS) and tested for solubility, uptake by THP-1 macrophages (uptake), AGE formation (AGE), glycation (OPA), percentage of α-helix (helix) or β-sheet (sheet), and exposure of hydrophobic regions (ANS). The aggregation-related parameters, proportion of monomer, oligomers and polymers are not shown as they did not differ between the samples. (PDF) [file pone.0236212.s005.pdf]
