# Supplementary material for: Hydrophobicity drives receptor-mediated uptake of heat-processed proteins by THP-1 macrophages and dendritic cells, but not cytokine responses
Source: PLoS One. 2020 Aug 14;15(8):e0236212. doi: 10.1371/journal.pone.0236212 (PMC7428126; doi:10.1371/journal.pone.0236212)
Supplement: S1 Table — (PDF) [file pone.0236212.s006.pdf]

S1 Table. LPS contamination in samples (ng/0.1 mg protein)

Treatment	Thyroglobulin	Lysozyme	BLG
L	0.69	0.43	0.00
L-glu	0.71	0.36	0.01
L-lac	0.89	0.34	0.02
L-gos	0.97	0.39	0.00
H	0.20	0.23	0.00
H-glu	-	-	0.00
H-lac	-	0.32	0.03
H-gos	0.03	0.17	0.02
W	0.90	0.17	0.00
W-glu	0.71	0.24	0.00
W-lac	0.84	0.30	0.01
W-gos	0.80	0.28	0.00
EDC	0.05	0.31	0.00
native	1.00	0.63	0.16
