# Supplementary material for: Hydrophobicity drives receptor-mediated uptake of heat-processed proteins by THP-1 macrophages and dendritic cells, but not cytokine responses
Source: PLoS One. 2020 Aug 14;15(8):e0236212. doi: 10.1371/journal.pone.0236212 (PMC7428126; doi:10.1371/journal.pone.0236212)
Supplement: S1 Protocol — (PDF) [file pone.0236212.s007.pdf]

S1 Protocol

- Lipopolysaccharide (LPS) detection

The concentration LPS in each sample was checked using the EndoZyme® Recombinant Factor C Assay (Hyglos GmbH, Bernried am Starnberger See, Germany) according to the manufacturer's instructions.
